# Determinants of life expectancy with unmet needs in the Chinese older population

**DOI:** 10.1007/s10433-026-00943-8

**Published:** 2026-08-13

**Authors:** Hui Wang, Bo Hu

**Affiliations:** 1 Institute for Population and Development Studies, School of Public Policy and Administration, Xi’an Jiaotong University, Xi’an, Shaanxi Province China; 2 Care Policy and Evaluation Centre (CPEC), London School of Economics and Political Science, London, UK

**Keywords:** Long-term care, Social disparities, Life expectancy, Unmet needs, Markov model

## Abstract

This study investigates the determinants of and disparities in life expectancy (LE) with unmet long-term care needs among older people in China. The data come from four waves of the China Health and Retirement Longitudinal Study (CHARLS), a nationally representative survey conducted between 2013 and 2020 (N = 42,480). Continuous time multi-state Markov modelling is combined with the life table approach to estimate the total life expectancy and life expectancy with met and unmet needs. Chinese older people aged 60 are expected to spend an average of 2.02 years with unmet care needs, comprising about one-third of their LE with care needs (6.44 years). LE with unmet needs is significantly longer among women, single people, older people with a lower level of education, and those living in rural areas. The duration of unmet needs over the remaining lifetime is estimated to be more than three times longer among the most disadvantaged group than the most advantaged group of older people (3.40 years vs 1.07 years). Life expectancy with unmet needs provides important information about older people’s quality of life and, like (un)healthy life expectancy, can be used for health economic evaluations and to monitor the performance of a health and care system. Targeted policies and interventions designed to support disadvantaged groups are essential to reduce the lifetime disparities in unmet needs.

**Supplementary Information:**

The online version contains supplementary material available at https://doi.org/10.1007/s10433-026-00943-8.

## Introduction

Long-term care is crucially important to people who in later life experience a decline in functional capability and need help to perform daily activities such as eating, dressing, and doing shopping. With continued global population ageing, demand for long-term care is expected to keep growing fast in the coming decades. It is a policy priority for many governments to make sure that sufficient care resources are made available to everyone who needs them, and that the provision of care can keep up with the demand. Unmet care needs have multiple consequences for older people’s lives including a decline in mental health, accelerated progression of functional disability, increased risks of falls, early admissions to care homes, and heightened mortality rates (He et al. 2015; Gaugler et al. 2005; Read et al. 2021; LaPlante et al. 2004).

There is an ongoing debate about the conceptualisation of unmet care needs. Existing research distinguishes between absolute and relative unmet needs (Vlachantoni 2019; Lima and Allen 2001). The former refers to a situation where an older adult needs help to perform certain activities of daily living but does not receive any help at all. It can be a useful measure when researchers want to make comparisons between different (sub-) populations (Allen et al. 2014; García-Gómez et al. 2015). The latter arises when older people with social care needs do receive help but are not fully satisfied with the help or need more help. Relative unmet need is a subjective measure and enables researchers to investigate whether a care need is undermet or fully met (Peng et al. 2015; Freedman and Spillman 2014).

Numerous studies have empirically investigated the factors associated with unmet care needs and identified considerable disparities in unmet needs among older people. First, the international literature has reported converging evidence that people who are single, live alone, or have weak family bonds are more likely to have unmet needs, pointing to the important role of social support networks in protecting older people from unmet needs (Lima and Allen 2001; Peng et al. 2015; Kröger et al. 2019; Dunatchik et al. 2019; Hu and Wei 2022). Second, people with a higher level of functional disability require more resources to fully meet their needs and thus face a higher risk of experiencing unmet needs (Kröger et al. 2019; Dunatchik et al. 2019; Brimblecombe et al. 2017). Moreover, an increasing number of studies have shown that unmet needs for social care are disproportionally concentrated among people with a lower socioeconomic status (García-Gómez et al. 2015; Albuquerque 2022). People with lower economic means often have a higher level of care needs and a lower level of affordability in regard to purchasing care from the market. Therefore, economic poverty and unmet needs are often entangled. Finally, research focussing on geographical variations has found that older people in Mediterranean countries are more likely to experience unmet needs than in Nordic, Eastern, and Western European countries (Calderón-Jaramillo and Zueras 2023), and that older people living in rural China are more likely than those in urban China to experience unmet needs (Zhu and Österle 2017; Zhu 2015).

Recent research has shown an elevated interest in the dynamics and longitudinal trends of unmet needs. Vlachantoni et al. (2024) found that individual circumstances in terms of unmet needs can change swiftly over time, which leads to diverse trajectories of unmet needs. Older people with a higher level of functional disability are more likely to experience a delay in fulfilling their care needs, while those without a strong support network face a higher risk of experiencing repeated unmet needs. Zhang (2025) conducted an age-period-cohort analysis of the Chinese older population and found that unmet needs are more pronounced among more recent cohorts than previous generations, especially in urban areas. Qian et al. (2023) looked at the prevalence of unmet needs in the older population between 2011 and 2018 among 31 countries. They noted that most countries showed a decreasing trend in the share of older people with a functional disability receiving assistance, which seems to indicate that the care gap is expanding and existing systems are struggling to meet the growing care demand. Echoing this, a study in England also reported a rise in the overall prevalence of unmet needs over time among older people living with dementia, but such an increase is attenuated among women and wealthier older people (Read et al. 2021).

## The present study

The present study investigates the duration of unmet care needs Chinese older people experience over their remaining lifetime. It concerns estimating the total number of years older people aged 60 spend with met and unmet long-term care needs. Older people in China heavily rely on and prefer family members, especially working-age children, to meet their care needs. This remains the case despite recent efforts of the Chinese government to pilot long-term care insurance schemes in multiple cities across the country (Pei et al. 2024). There are currently 304 million older people aged 60 and over in China, which is projected to increase by 54% to 468 million by 2045. In comparison, the number of working-age adults aged 20–59 is projected to decrease by 17%, from 808 million in 2025 to 669 million in 2045 (United Nations 2024). With such a dramatic demographic shift in the horizon, addressing unmet needs in the older population will continue to be a critical issue in China. The existing studies on unmet needs, as reviewed in the previous section, have focussed on the prevalence and incidence of unmet needs in various groups of the older population at fixed points in time. Little effort has been devoted to investigating unmet needs as an enduring experience for the remainder of an older person’s life. The lack of research and evidence precludes a rigorous debate about how to protect older people from lifelong experiences of unmet needs.

Life course theory suggests that risk factors such as poor health, socioeconomic disadvantage, instability in social networks, and inadequate support interact with each other and create feedback loops in life outcomes over time (Pearlin 2010). Such a dynamic process is expected to be salient in older people’s experience of unmet needs, because unmet needs are shaped by prior disadvantage and may also influence later life health and care trajectories. As discussed above, people living in rural China and those with a lower socioeconomic status are more likely to have poor health and heightened care needs, which increases their risk of unmet needs. In turn, unmet needs can accelerate the progression of illness and functional limitations and incur further care expenditure while draining economic resources (Huang et al. 2024). Unmet needs and their risk factors not only accumulate but also reinforce each other over time. Therefore, we expect that unmet needs among different groups of older population may widen across later life and be manifested as significant disparities in the number of remaining years lived with unmet needs.

Healthy life expectancy is a metric that combines the quantity and quality of life, reflecting the total number of years individuals are expected to live in good health (Stiefel et al. 2010). Life expectancies with and without functional disabilities are often viewed as indicators of life expectancies with and without long-term care needs, respectively (Kingston et al. 2022, 2018). It has been widely agreed that more health and public policy efforts should be geared towards disability compression. This is to maximise the period of life spent in good functional capability while minimising the duration of time spent in disability or with long-term care needs (Crimmins 2015). We argue in this study that when people do have care needs, the number of years they spend with or without care and support also matters. Life expectancy with unmet needs can be another useful metric for health economic evaluations and care planning.

## Methods

### Data

The data in this study came from the China Health and Retirement Longitudinal Study (CHARLS), which provides comprehensive information on the social-demographic background, health conditions, and family characteristics of the community-dwelling population aged 45 years old and above. CHARLS is a nationally representative survey using a longitudinal design and multistage stratified probability-proportionate-to-size sampling to collect data from 150 counties or districts and 450 villages or urban communities across 28 provinces in China. The baseline survey was conducted in 2011 with follow-up surveys in 2013, 2015, 2018, and 2020, respectively. Refreshed samples were collected in the follow-up surveys to maintain the representativeness of the sample (Zhao et al. 2020).

This study drew on the later four waves of CHARLS data collected between 2013 and 2020. CHARLS 2011 data were not included in our analysis because the questions relating to long-term care receipt in that wave were different from those in later waves, which led to an inconsistent measure of unmet care needs. There were 80,977 person-observations across the four waves. We excluded people aged below 60 years old or with missing information on age in each wave. This left us with 42,480 individuals (i.e., person-observations) aged 60 and over across the four waves, which was our final analytical sample (Supplementary Fig. A1).

### Measures

#### Care needs and unmet needs

Following previous studies, we used functional disability to measure long-term care needs (Kingston et al. 2022). The CHARLS asked participants whether they could perform five activities of daily living (ADLs: dressing, bathing, eating, moving, and using the toilet) and five instrumental activities of daily living (IADLs: doing housework, cooking, taking medication, shopping, and managing money). ADLs relate to personal care tasks, while IADLs relate to domestic care tasks (Kröger et al. 2019). There were four options: ‘I have no difficulty doing it,’ ‘I have difficulty but can still do it myself,’ ‘I need help,’ and ‘I cannot do it.’ Consistent with previous studies (Zhu and Österle 2017), the latter two options were treated as having an ADL or IADL care need.

Unmet care needs in each wave were measured by combining whether participants had care needs with whether they received care from family members or paid help for each task. Participants were considered to have no care needs if they reported neither IADL nor ADL care needs. Participants were considered to have their care needs met if they had any IADL or ADL care needs and received all the corresponding help. Having any care needs but not receiving help with at least one ADL or IADL task was considered having unmet needs. People with unmet needs may have unmet ADL needs only (i.e. unmet personal care needs), unmet IADL needs only (i.e. unmet domestic care needs), or both. The proportion of people with both unmet ADL needs and unmet IADL needs is small (around 0.85%), so we combined them with people having unmet ADL needs only. We ended up with four mutually exclusive groups of older people: no care needs, met care needs, unmet IADL needs only, and unmet ADL needs (with or without unmet IADL needs) (Supplementary Fig. A2). This means that we adopted an absolute approach to measuring unmet needs. The CHARLS questionnaire did not ask care recipients whether they were satisfied with help or need more help, which prevented us from taking a relative approach in this study.

#### Identifiers of population subgroups

To examine disparities in LE with unmet needs in the older population, we divided the sample into subgroups according to individuals’ socio-demographic, socioeconomic, and geographical factors. They include gender (men, women), education (primary education or below, secondary education or above), marital status and living arrangements (married or cohabiting with a partner, single people living with children or other family members, single people living alone), residence (rural, urban), and region. Single people include those who were separated, divorced, widowed, or never married. Following the method of the National Bureau of Statistics (2011), we used the zip code information derived from each respondent’s unique ID to classify the sample into four geographic regions: East China, Central China, Northeast China, and West China.

### Statistical analysis

**Fig. 1:**
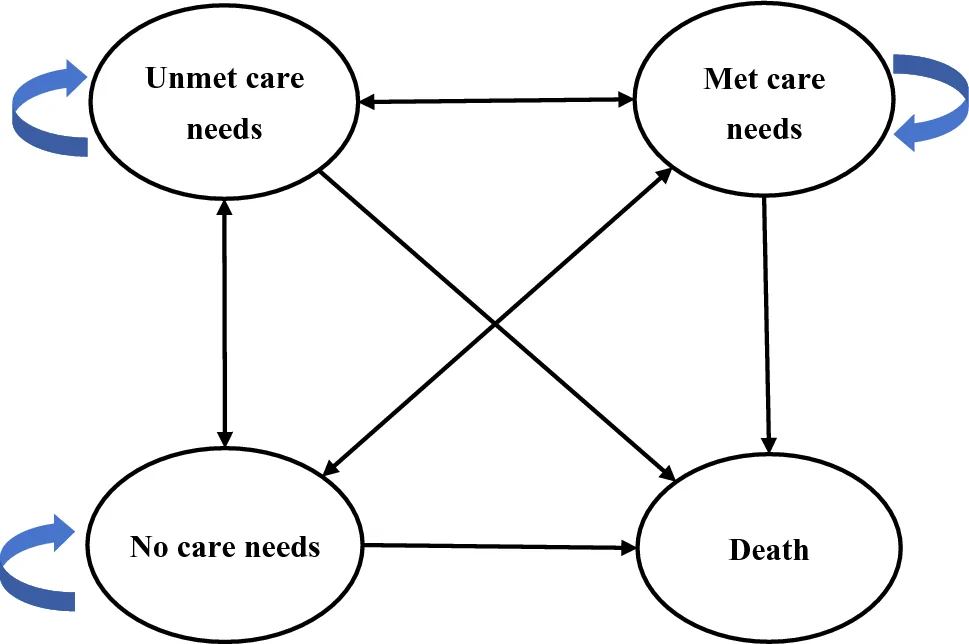
Four-state continuous time Markov model of met and unmet needs. *Note*: Permitted transitions are shown with arrows

The analyses consisted of two steps. In the first step, we constructed a continuous time Markov model to estimate the transition probabilities between four states: no care needs, fully met needs, unmet needs, and death. Death was treated as an absorbing state (Fig. 1). Such a model requires information on the exact date of death. This piece of information is available in CHARLS 2013 and CHARLS 2020, but not in CHARLS 2015 and 2018. In the latter case, we followed prior studies to treat the midpoint between the wave reporting the death and the date of the interview in the previous wave as the imputed date of death (Li et al. 2024; Talifu et al. 2024). Age is the key predictor in the Markov model. Subgroup identifiers were included in the Markov model as covariates. Gender and education were included as time-invariant covariates while the other variables were included as time-varying covariates in the models. Transition probability matrices were estimated for the overall sample and subpopulations by gender, education, marital status and living arrangements, residence, and region. The *R* package *msm* was used to estimate the transition probabilities (Jackson 2011). The maximum likelihood estimation approach was used.

In the second step, we followed the approach proposed by van den Hout et al. (2019) to estimate the number of years people spent in three alive states. This involves first solving the following integral to estimate the conditional life expectancy in state s at age t+u conditional upon state r at age t and given a vector of covariates x:

1 $$LE_{{{\mathrm{rs}}}} \left( {t|x} \right) = \mathop \smallint \limits_{0}^{\infty } P(Y_{t + u} = s|Y_{t} = r,x)du$$

where $P(Y_{t + u} = s|Y_{t} = r,x)$ denotes the transition probability between age t and age t+u, which was derived from the Markov model in the first step. Then the marginal life expectancy in state s $({LE}_{s})$ was calculated as follows:

2 $$LE_{s} = \mathop \sum \limits_{r = 1}^{3} P(Y_{t} = r|x)LE_{rs} (t|x)$$

where $P(Y_{t} = r|x)$ denotes the probability in state r at age t and was estimated using a multinomial logistic regression model. Marginal life expectancy is the life expectancy in a state irrespective of the initial state at age t and is what we are interested in. Eq. (2) gave us the LE with no care needs, LE with met care needs, and LE with unmet needs. Adding up the three marginal LEs gave us the total life expectancy (TLE) at the age of 60.

The 95% confidence intervals of LEs were estimated using a simulation approach: 1000 sets of random numbers were repeatedly drawn from a multivariate normal distribution. The maximum age was set at 105 years because the CHARLS data shows that the probability of survival beyond that age is negligible. The *R* package *elect* was used for this step of the analysis.

We further broke down LE with unmet needs into LE with unmet IADL needs only and LE with unmet ADL needs. A continuous time Markov model failed to converge due to the small number of people transitioning between unmet IADL needs only and unmet ADL needs in the CHARLS sample (Supplementary Table A1). Therefore, we constructed a discrete time multi-state life table to estimate, within the LE with unmet needs, the proportions of time spent with unmet ADL needs and unmet IADL needs only, respectively (Preston et al. 2000). Applying these proportions to the total number of years with unmet needs derived from Eq. (2) gave us the central estimates and the 95% CIs of life expectancies with unmet ADL needs and unmet IADL needs only. Data and formulae used in this step of calculation are reported in the Supplementary materials (see Tables A2 and A3).

We calculated the differences in LEs with unmet ADL/IADL needs between the subgroups of the population to investigate social disparities. The 95% CIs of the differences in life expectancies were derived using the following formula (Li et al. 2024):

$$95\% \;CI = {{{\mathrm{diff}}}}_{{{\mathrm{LE}}}} \pm 1.96\sqrt {{\mathrm{var}} \left( {LE_{{{\mathrm{high}}}} } \right) + {\mathrm{var}} \left( {LE_{{{\mathrm{low}}}} } \right)}$$

where ${{{\mathrm{diff}}}}_{\mathrm{L}\mathrm{E}}$ refers to the difference in the central estimates of LE between the two groups of people, and ${{{\mathrm{var}}}}\left({\mathrm{L}\mathrm{E}}_{{{\mathrm{high}}}}\right)$ and ${{{\mathrm{var}}}}\left({\mathrm{L}\mathrm{E}}_{{{\mathrm{low}}}}\right)$ represent the variance in LE for the high and low LE groups, respectively. The number of missing values in the dataset is negligible (Supplementary Table A4). We dropped all observations with missing values in the analyses.

## Results

### Life expectancy with unmet needs: entire sample

Table 1 shows the characteristics of the entire analytical sample. In 2013, 77.1% of the sample had no long-term care needs, 15.1% had their care needs met, 4.9% had unmet IADL needs only, and 2.9% had unmet ADL needs. The proportion of people with unmet care needs fluctuated between 2013 and 2020. The average age of the sample was 68.9 years old in 2013, which increased to 70.6 years old in 2020. Women accounted for 50.3% of the sample in 2013, with this figure increasing to 51.2% in 2020. In 2013, 19.9% of older people aged 60 and over received secondary education or above, which increased to 26.6% in 2020. The proportion of married people decreased from 78.7% in 2013 to 76.1% in 2020. The proportion of single people living alone increased from 8.1% in 2013 to 11.1% in 2020. More than 60% of older people lived in rural areas. Around one-third lived in West China, nearly 30% lived in Central China, and around 7% lived in Northeast China.

**Table 1:** Characteristics of the analytic sample

	2013	2015	2018	2020
	n = 8439	n = 10,233	n = 11,588	n = 11,990
*States of care needs*				
No care needs	77.14%	76.28%	74.91%	78.96%
Met care needs	15.12%	15.15%	16.90%	15.00%
Unmet IADL needs only	4.85%	4.86%	5.54%	4.30%
Unmet ADL needs	2.89%	3.71%	2.65%	1.74%
Mean age (standard error)	68.86(7.01)	69.22(7.24)	70.05(7.42)	70.62(7.40)
*Age group*				
60–64	37.58%	35.48%	30.28%	25.24%
65–69	24.99%	25.93%	27.74%	29.08%
70–74	17.50%	17.38%	17.84%	20.48%
75–79	11.44%	11.57%	12.28%	12.69%
> = 80	8.50%	9.64%	11.86%	12.52%
*Gender*				
Female	50.29%	50.32%	50.37%	51.19%
Male	49.71%	49.68%	49.63%	48.81%
*Education*				
Primary education or below	80.15%	79.63%	76.47%	73.45%
Middle education or above	19.85%	20.37%	23.53%	26.55%
*Marital status and living arrangements*				
Married or cohabiting	78.68%	78.59%	77.92%	76.14%
Single & living with others	13.18%	13.64%	13.21%	12.72%
Single & living alone	8.14%	7.78%	8.87%	11.14%
*Rural–urban residents*				
Urban	39.77%	39.65%	39.43%	34.33%
Rural	60.23%	60.35%	60.57%	65.67%
*Region*				
East China	30.67%	31.18%	31.19%	32.52%
Central China	28.58%	28.55%	28.68%	29.12%
Northeast China	6.94%	6.84%	6.71%	6.31%
West China	33.71%	33.42%	33.41%	32.04%

Table 2 shows the overall TLE and LE with met and unmet needs of Chinese older people. People at the age of 60 are expected to live an average of 21.4 years (95% CI 21.0–21.7). Our estimates of the TLE of Chinese older people using the CHARLS data are very close to the statistics published by the United Nations (2024) (Supplementary Table A5). The number of years Chinese older people spent without and with long-term care needs is 15.0 (95% CI 14.7–15.2) and 6.4 (95% CI 6.2–6.7), respectively (Table 2). For the 6.4 years of life with care needs, people spent 4.4 years (95% CI 4.2–4.6) with their care needs met, and LE with unmet care needs is estimated to be 2.0 years (95% CI 1.9–2.1).

**Table 2:** Life expectancy with unmet and met care needs among Chinese older people aged 60, central estimates and 95% confidence intervals

Life expectancy (years)					
	No care needs	Unmet IADL needs only	Unmet ADL needs	Met care needs	Total
Total	14.98(14.65, 15.22)	1.32(1.24, 1.39)	0.70(0.66, 0.74)	4.42(4.21, 4.59)	21.42(20.95, 21.74)
*Gender*					
Men	15.21(14.21, 15.50)	0.98(0.74, 1.05)	0.49(0.34, 0.53)	3.21(2.70, 3.37)	19.89(18.13, 20.22)
Women	14.72(14.24, 15.04)	1.73(1.58, 1.85)	0.92(0.84, 0.98)	5.82(5.44, 6.09)	23.18(22.42, 23.61)
*Education*					
Higher education	17.72(16.64, 18.44)	0.82(0.57, 0.96)	0.53(0.37, 0.62)	4.03(3.45, 4.52)	23.10(21.44, 24.05)
Lower education	14.42(14.07, 14.67)	1.42(1.33, 1.50)	0.73(0.68, 0.77)	4.54(4.31, 4.75)	21.11(20.56, 21.48)
*Marital status and living arrangements*					
Married or cohabiting	15.30(14.88, 15.62)	1.16(1.06, 1.25)	0.64(0.58, 0.69)	4.76(4.42, 4.99)	21.86(21.18, 22.31)
Single and living with others	13.04(12.22, 13.64)	1.66(1.42, 1.85)	0.79(0.67, 0.88)	4.23(3.73, 4.60)	19.72(18.43, 20.45)
Single and living alone	15.06(13.94, 15.80)	1.66(1.36, 1.85)	0.98(0.80, 1.09)	3.63(3.04, 4.02)	21.32(19.54, 22.22)
*Rural/Urban residence*					
Urban residents	16.15(15.57, 16.56)	1.09(0.97, 1.20)	0.59(0.53, 0.65)	4.27(3.90, 4.54)	22.10 (21.30, 22.59)
Rural residents	14.27(13.86, 14.56)	1.47(1.34, 1.56)	0.75(0.69, 0.80)	4.52(4.23, 4.72)	21.01 (20.35, 21.40)
*Region*					
East China	15.94(15.23, 16.38)	1.31(1.15, 1.43)	0.60(0.53, 0.65)	4.34(3.94, 4.63)	22.20(21.13, 22.76)
Central China	14.47(13.89, 14.92)	1.43(1.27, 1.56)	0.73(0.65, 0.80)	4.37(4.01, 4.65)	20.99(20.16, 21.54)
Northeast China	14.47(12.87, 15.48)	1.25(0.83, 1.53)	0.71(0.47, 0.87)	4.95(3.79, 5.51)	21.32(18.55, 22.48)
West China	14.62(14.04, 15.03)	1.31(1.14, 1.42)	0.72(0.63, 0.78)	4.43(4.04, 4.71)	21.09(20.06, 21.60)
*Multi-attribute groups*					
Lowest LE with unmet needs	17.63(16.48, 19.46)	0.67(0.45, 0.80)	0.40(0.27, 0.48)	3.79(2.83, 4.22)	22.49(20.81, 23.86)
Highest LE with unmet needs	12.94(12.00, 13.50)	2.18(1.92, 2.38)	1.22(1.07, 1.33)	5.07(4.46, 5.41)	21.41(20.04, 22.07)

Lowest LE with unmet needs: older people who were men, married or cohabiting, with higher levels of education, and living in urban areas; Highest LE with unmet needs: older people who were women, single, with lower levels of education, and living in rural areas

**Fig. 2:**
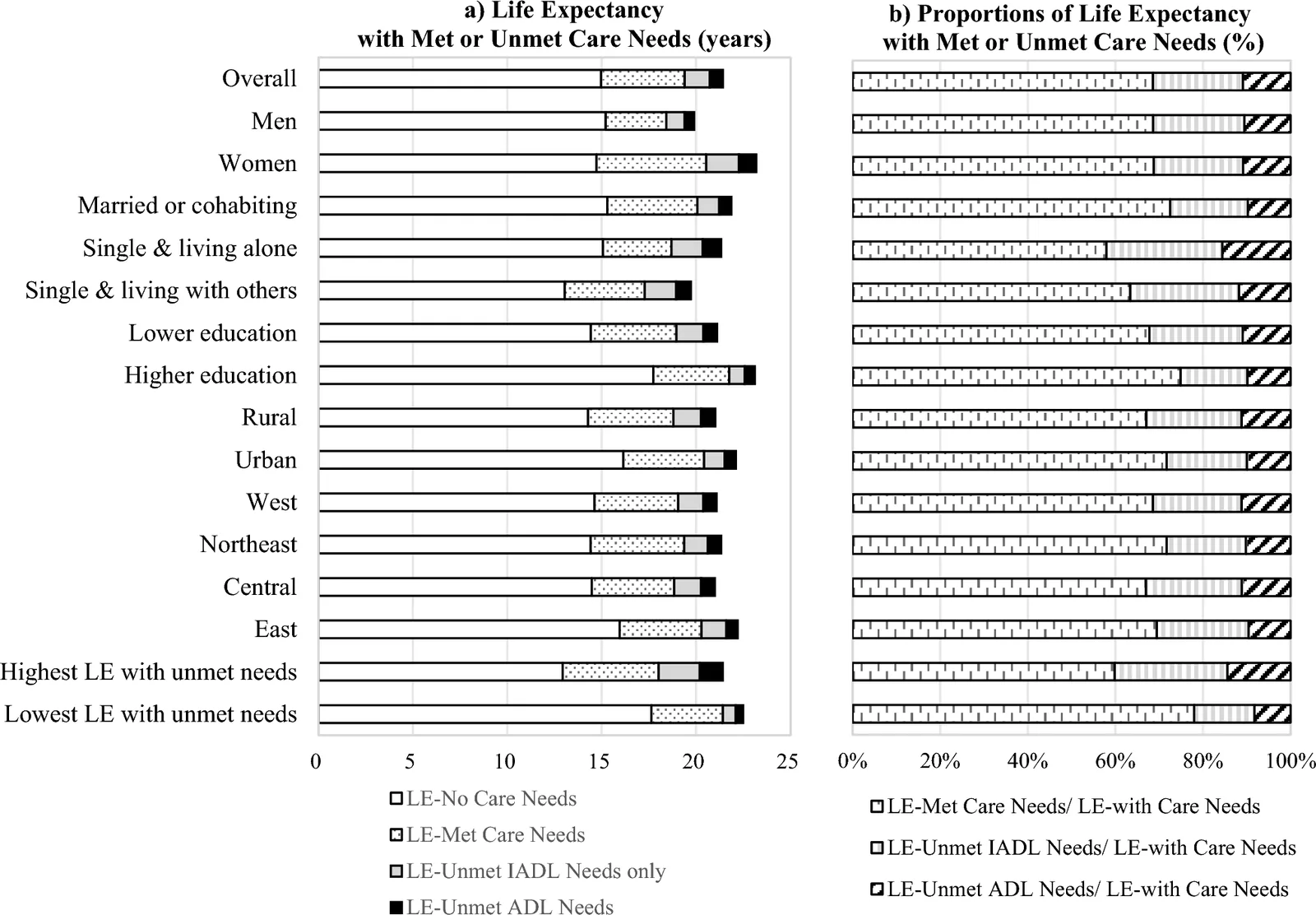
Life Expectancy (LE) with met and unmet care needs according to social groups

LE with unmet needs estimated using the approach of van den Hout et al. (2019) is very close to that obtained from the discrete-time life table. In most cases, the differences between the two sets of estimates are no more than 3% (Supplementary Table A2). This high degree of consistency provides a basis for combining the two approaches to derive LE with unmet IADL needs only and LE with ADL needs and their respective 95% CIs, as presented in Table 2 and Supplementary Table A3. Among the 2.0 years with unmet needs, people spent 1.3 years (95% CI 1.2–1.4) with unmet IADL needs only and 0.7 years (95% CI 0.66–0.74) with unmet ADL needs. Put differently, during the 6.4 years when people had care needs, 21% of the time was spent with unmet IADL needs, and 11% was spent with unmet ADL needs (Panel b, Fig. 2).

We conducted further analyses to check the robustness of the results. First, we expanded the analytical sample to respondents aged 50 and over to account for left censoring. The estimated LE with unmet needs among people at the age of 60 changed very little (Supplementary Table A6). Second, logistic regression analysis shows that the risk of unmet needs did not differ significantly between respondents staying in the survey and those lost to follow-up, suggesting that any potential selection bias associated with sample attrition should be negligible (Supplementary Table A7).

We conducted sensitivity analyses using alternative cut-off points to define unmet needs and recalculated life expectancy with unmet needs accordingly (Supplementary Fig. A3). Defining unmet needs as receiving no help with more than one (instead of at least one) ADL/IADL task leads to a notable reduction in the number of years spent with unmet needs. LE with unmet needs also varies markedly across specific ADL or IADL tasks. An overall pattern is that the proportion of LE spent with unmet needs is higher for ADL tasks than for IADL tasks.

### Life expectancy with unmet needs: subgroups

Figure 2 shows the estimates and proportions of LEs with met and unmet care needs across subgroups of older people at the age of 60. The LE with care needs at the age of 60 was much higher among women than men (8.5 vs 4.7 years). The same can be said about LE with unmet needs. Women spent more years with unmet IADL needs (1.7 vs 1.0 years) and unmet ADL needs (0.9 vs 0.5 years) than men. Despite the gender differences in the absolute number of years, the proportion of life years spent with unmet needs was similar (31.41% vs 31.29%) between men and women (Panel b, Fig. 2).

Regarding marital status and living arrangements, married or cohabiting people at the age of 60 spent fewer years with unmet IADL needs (1.2 vs 1.7 years) and unmet ADL needs (0.6 vs 1.0 years) than single people living alone. In those years with care needs, the proportion of time with unmet needs was the lowest among married or cohabiting people (27.4%), followed by single people living with others (36.7%), and was the highest among single people living alone (42.1%) (Panel b, Fig. 2).

For less well-educated people, the duration of unmet IADL needs at the age of 60 was 1.4 years and that of unmet ADL needs was 0.7 years, which are 73.2% and 37.7% higher, respectively, than the figures for better-educated people. Conditional upon having care needs, less well-educated people spent nearly one-third of the time (32.1%) with unmet needs, whereas better-educated people spent one-fourth of the time (25.1%) with unmet needs.

For rural residents, the duration of unmet IADL needs at the age of 60 was 1.5 years and that of unmet ADL needs was 1.1 years, which are 34.9% and 27.1% higher, respectively, than the figures for urban residents. Conditional upon having care needs, the proportion of time with unmet needs was 32.9% among rural residents and 28.2% among urban residents. LE with unmet needs was slightly lower in both absolute and relative terms among people in Northeast China and East China than in Central China and West China.

We investigated the boundaries of LE with unmet needs by focussing on two contrasting subgroups characterised by a combination of the attributes discussed above. The first one relates to married or cohabiting men aged 60, who received better education and lived in urban areas. This group had the lowest LE with unmet needs at the age of 60, representing the most advantaged group in terms of care access in our sample: LE with unmet IADL needs was 0.7 years, and that with unmet ADL needs was 0.4 years. The other group concerns those with the highest LE with unmet needs, representing the most disadvantaged group in terms of care access: single women aged 60, who were less well-educated and lived in rural areas. People in this group were expected to live 2.2 years with unmet IADL care needs and 1.2 years with unmet ADL care needs (Table 2). During years with care needs, the proportion of time spent with unmet care needs was 22.0% among people in the most advantaged group, which stands in contrast to 40.1% among people in the most disadvantaged group (Panel b, Fig. 2).

**Fig. 3:**
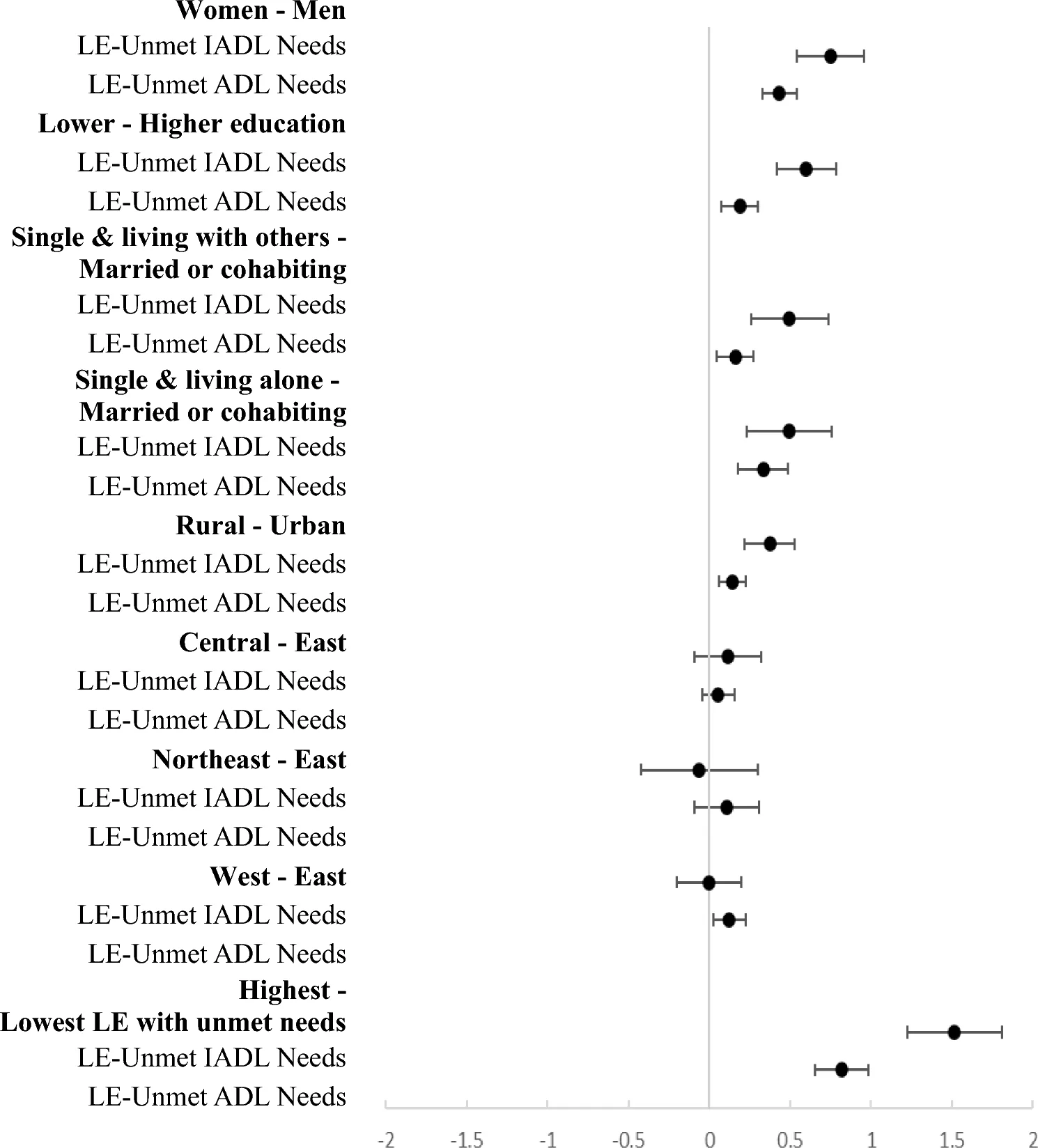
Differences in life expectancy with unmet needs between subgroups of older population aged 60 years. *Note*: The difference between group a and group b is calculated as group a minus group b. Lowest LE with unmet needs: older people who were men, married or cohabiting, with higher levels of education, and living in urban areas; Highest LE with unmet needs: older people who were women, single, with lower levels of education, and living in rural areas

Figure 3 reports the differences in LEs with unmet needs between different groups of older people. The underlying data are reported in Supplementary Table A8. LEs with unmet IADL needs and unmet ADL care needs are 0.75 years (95% CI 0.55–0.96) and 0.43 years (95% CI 0.33–0.54) longer, respectively, among women than men. LEs with unmet IADL needs and unmet ADL needs are 0.60 years (95% CI 0.26–0.74) and 0.19 years (95% CI 0.08–0.30) longer, respectively, among less well-educated than better-educated people. LEs with unmet IADL needs and unmet ADL needs are 0.49 years (95% CI 0.26–0.74) and 0.33 years (95% CI 0.18–0.49) longer, respectively, among single people living alone than married people. LEs with unmet IADL care needs and unmet ADL care needs are 0.37 years (95% CI 0.22–0.53) and 0.14 years (95% CI 0.06–0.22) longer, respectively, among rural than urban residents. For people in the most disadvantaged group, their LEs with unmet IADL care needs and unmet ADL care needs are 1.51 years (95% CI 1.22–1.81) and 0.82 years (95% CI 0.65–0.99) longer, respectively, than LEs with unmet needs for people in the most advantaged group.

## Discussion

This paper estimated the life expectancy with unmet needs of Chinese older people at the age of 60 and investigated the extent and drivers of its disparities. Older people aged 60 years have a life expectancy of 21.4 years, with 30% of this time (6.4 years) spent with care needs. The life expectancy with unmet needs amounts to 2.0 years, constituting almost one-third of the total number of years spent with care needs. Older people aged 60 years are expected to experience an average of one year with unmet needs for every 11 years of their remaining life.

We found marked disparities in life expectancy with unmet needs in the Chinese older population. Women aged 60 spent 1.2 more years with unmet needs than men, which is due to women’s longer TLE and LE with care needs. The duration of unmet needs over older people’s remaining lifetime is 47% longer among single people living alone than married or cohabiting people. Such disparity not only highlights the importance of care and support from family members in a country like China where older people rely heavily upon family care to meet their needs but also points to the urgency and necessity for the government to step up support for older people, especially for those who have limited or no access to family care (Hu and Wittenberg 2025).

Life expectancy with unmet needs is inversely correlated with educational achievements. We found that people with lower education spent 60% more time with unmet needs than those with higher education. This resonates with the evidence reported in the international literature that the prevalence of unmet needs is higher among people with lower socioeconomic status (Vlachantoni 2019; Kröger et al. 2019; Zhu 2015; Qian et al. 2023). Geographical disparities are mainly reflected in rural–urban differences, with rural residents spending 30% more time in their later life with unmet needs than urban residents. Chinese older people living in rural areas spend more time with unmet needs for two reasons. First, there are a large number of working-age migrant workers each year moving from rural to urban areas to pursue better job opportunities, which reduces access to family care in rural China (Zhu and Österle 2017). Second, due to unbalanced economic development, formal care services are either scarce with low service quality or beyond older people’s affordability in rural China (Feng et al. 2020).

We did not find strong evidence that life expectancy with unmet needs varies substantially across the four regions of China. This is probably because the factors associated with unmet needs such as socio-demographic characteristics and socioeconomic status vary across the four regions, and their impacts on the LE with unmet needs offset each other. However, we did observe that disparities in LE with unmet needs tend to be cumulative. For people in the most disadvantaged group, the duration of unmet needs over their remaining lifetime is 3.2 times longer than that for the most advantaged group. After adjusting for age, gender and non-response weights, people with the lowest and highest LE with unmet needs account for 9.8% and 8.7% of the CHARLS sample, respectively. These translate into 25.87 million and 22.97 million Chinese older people, respectively. These two groups may represent older people with extreme experiences of unmet needs, but they are by no means rare cases in the population.

Our study brings two novel contributions to the existing literature. First, numerous studies have looked at the prevalence and incidence of unmet needs, but little research has been done to investigate the longevity of unmet needs. Our analyses demonstrate that unmet needs are not passing or transient events. Instead, they can be sustained, lived experiences that span a significant part of older adults’ life journeys and differ greatly according to people’s social identities, resources, and geographical locations. Unmet care needs, like other life outcomes such as health conditions and socioeconomic positions, can accumulate over time and result in lifetime inequalities. Indeed, disparities in unmet needs are both an outcome of the distribution and re-distribution of care resources and a process of production and reproduction of social (dis)advantages.

Second, our study shows that it is meaningful to distinguish between the time people spend with met and unmet needs because of the negative and long-lasting impacts of unmet needs on people’s well-being and health outcomes. Life expectancy with unmet needs is a useful extension to the concepts of healthy (and unhealthy) life expectancy. It can be a powerful tool for identifying long-term care disparities in society and for monitoring the performance of a health system. Moreover, it enriches the existing theoretical framework of health economic evaluations: success in interventions should be measured not only by extending LE with good health but also by reducing LE with unmet needs.

Given the marked disparities in LE with unmet needs identified in this study, we call for policymakers to pay more attention to policies and interventions that can maximise the period of time when older people can access adequate personal and domestic care and minimise the duration of unmet needs. The Chinese government has recently announced the nationwide rollout of the long-term care insurance (LTCI). Policymakers may want to consider tailoring the eligibility criteria and benefit structure of LTCI in different localities to make sure older people with varied support networks and socioeconomic conditions receive adequate support. Despite the expansion of government support, family members will remain the primary caregivers in many Chinese households. For older people with severe functional limitations, family caregivers are at a higher risk of burnout. Government support for family caregivers such as respite services and flexible working arrangements remains uncommon in Chinese society (Zhao et al. 2022). Yet it will be essential for sustaining caregivers’ capability and resilience, which will play a key role in reducing life expectancy with unmet needs among older adults.

This study focussed on people experiencing functional disabilities in later life, but our analytical framework would also be suitable to look at people with other types of care needs across the entire life span, such as mental illness and cognitive impairment. In addition, national surveys conducted in European countries and in the US include similar questions about long-term care needs and receipt of care (Lee et al. 2022). LE with unmet needs can be similarly estimated in those contexts. Discussions around the identification of, and reduction in the disparities in LE with unmet needs will be equally meaningful given the global trend of population ageing and rising demand for long-term care.

The limitations of this study should be duly acknowledged. First, due to data availability, we were only able to investigate absolute unmet needs but not relative unmet needs. For people receiving support, we were not able to further distinguish between LE with fully met and undermet needs. Second, we adopted the discrete-time life table approach in this study to disaggregate the LE with unmet needs into ADL and IADL needs. As more waves of CHARLS data become available in the future, a larger sample size will help solve the convergence problem in a continuous time model. Third, due to the large number of missing values in the income and wealth variables, we included education as the only socioeconomic variable.

## Conclusion

The rapidly ageing population and increasing life expectancy pose great challenges in addressing unmet long-term care needs and associated disparities among older people. Our findings indicate that the existing care resources and services remain insufficient to meet the rapidly growing care needs in China, with people spending a sustained period of time with unmet needs in later life. Moreover, life expectancy with unmet needs shows considerable disparities across various social groups. To promote healthy ageing and social equality, national policies and interventions should aim at effectively improving older people’s access to health and care resources, with a strong emphasis on reallocating resources towards those disadvantaged groups with a higher risk of unmet needs. Further evidence on life expectancy with unmet needs from other countries would provide valuable insights for capturing and comparing the progress of healthy ageing globally.

## Supplementary Information

Below is the link to the electronic supplementary material.

Supplementary file1 (DOCX 82 KB)

## Data Availability

The data are publicly available and can be found on the CHARLS website: http://charls.pku.edu.cn/en/.
