# Supplementary material for: Determinants of life expectancy with unmet needs in the Chinese older population
Source: Eur J Ageing. 2026 Aug 13;23(1):45. doi: 10.1007/s10433-026-00943-8 (PMC13759852; doi:10.1007/s10433-026-00943-8)
Supplement: Supplementary file 1 [file 10433_2026_943_MOESM1_ESM.docx]

**Supplementary materials**

**Supplementary Table A1** Counts of Observed Transitions between States of Care Needs in the Analytical Sample of CHARLS (2013**–**2020)

| To  From | **No Care Needs** | **Unmet IADL Needs only** | **Unmet ADL Needs** | **Met Care Needs** | | **Death** |
| --- | --- | --- | --- | --- | --- | --- |
| **No Care Needs** | 17087 | 788 | 344 | 2057 | 821 | |
| **Unmet IADL Needs only** | 607 | 157 | 83 | 391 | 176 | |
| **Unmet ADL Needs** | 292 | 85 | 101 | 252 | 121 | |
| **Met Care Needs** | 1439 | 350 | 213 | 1647 | 743 | |

**Supplementary Table A2** Life Expectancy with Unmet Needs: A Comparison of Two Approaches

|  | **Life Expectancy with unmet needs** | | |
| --- | --- | --- | --- |
|  | **ven den Hout et al.’s (2019) approach** | **Discrete-time Life Table** | **Difference** |
| **Age** |  |  |  |
| 60 | 2.03 | 1.97 | 3.0% |
| 65 | 1.87 | 1.82 | 2.7% |
| 70 | 1.68 | 1.64 | 2.4% |
| 75 | 1.46 | 1.44 | 1.4% |
| 80 | 1.24 | 1.22 | 1.6% |
| 85 | 0.99 | 1.10 | -10.0% |
| **Gender** |  |  |  |
| Men | 1.47 | 1.43 | 2.8% |
| Women | 2.64 | 2.57 | 2.7% |
| **Education** |  |  |  |
| Higher education | 1.35 | 1.34 | 0.7% |
| Lower education | 2.16 | 2.12 | 1.9% |
| **Marital status & living arrangements** | | | |
| Married or cohabiting | 1.80 | 1.88 | -4.3% |
| Single & living with others | 2.44 | 2.50 | -2.4% |
| Single & living alone | 2.63 | 2.62 | 0.4% |
| **Residence** |  |  |  |
| Urban residents | 1.69 | 1.65 | 2.4% |
| Rural residents | 2.22 | 2.17 | 2.3% |
| **Region** |  |  |  |
| East | 1.91 | 1.84 | 3.8% |
| Central | 2.15 | 1.94 | 10.8% |
| Northeast | 1.95 | 1.97 | -1.0% |
| West | 2.03 | 2.08 | -2.4% |

**Supplementary Table A3** Breaking down LE with Unmet Needs into LE with Unmet IADL needs only and LE with unmet ADL needs

|  | Discrete-time multi-state life table | | | | ven den Hout et al.’s (2019) approach | **Breaking down LE with unmet needs** | |
| --- | --- | --- | --- | --- | --- | --- | --- |
|  | LE-Unmet IADL Needs Only  (a) | LE-Unmet ADL Needs  (b) | (a)/((a)+(b))  (c) | (b)/((a)+(b))  (d) | LE-Unmet needs  (e) | **LE-Unmet**  **IADL Needs Only**  **(f)= (e) * (c)** | **LE-Unmet**  **ADL Needs**  **(g)= (e) * (d)** |
| Total | 1.29 | 0.68 | 65.39% | 34.61% | 2.03(1.89, 2.13) | 1.32(1.24, 1.39) | 0.70(0.66, 0.74) |
| Gender |  |  |  |  |  |  |  |
| Men | 0.95 | 0.48 | 66.35% | 33.65% | 1.47(1.11, 1.58) | 0.98(0.71, 0.97) | 0.49(0.36, 0.49) |
| Women | 1.68 | 0.89 | 65.33% | 34.67% | 2.64(2.42, 2.83) | 1.73(1.58, 1.85) | 0.92(0.84, 0.98) |
| Education |  |  |  |  |  |  |  |
| Higher education | 0.81 | 0.52 | 60.83% | 39.17% | 1.35(1.05, 1.58) | 0.82(0.57, 0.96) | 0.53(0.37, 0.62) |
| Lower education | 1.40 | 0.72 | 65.99% | 34.01% | 2.16(2.01, 2.27) | 1.42(1.33, 1.50) | 0.73(0.68, 0.77) |
| Marital status & living arrangements |  |  |  |  |  |  |  |
| Married or cohabiting | 1.21 | 0.67 | 64.43% | 35.57% | 1.80(1.64, 1.94) | 1.16(1.06, 1.25) | 0.64(0.58, 0.69) |
| Single & living with others | 1.69 | 0.80 | 67.87% | 32.13% | 2.44(2.10, 2.73) | 1.66(1.42, 1.85) | 0.79(0.67, 0.88) |
| Single & living alone | 1.65 | 0.97 | 62.93% | 37.07% | 2.63(2.16, 2.94) | 1.66(1.36, 1.85) | 0.98(0.98, 1.09) |
| Residence |  |  |  |  |  |  |  |
| Urban residents | 1.07 | 0.58 | 64.86% | 35.14% | 1.69(1.49, 1.84) | 1.09(0.97, 1.20) | 0.59(0.53, 0.65) |
| Rural residents | 1.43 | 0.73 | 66.07% | 33.93% | 2.22(2.03, 2.35) | 1.47(1.34, 1.56) | 0.75(0.69, 0.80) |
| Region |  |  |  |  |  |  |  |
| East China | 1.27 | 0.58 | 68.65% | 31.35% | 1.91(1.70, 2.08) | 1.31(1.15, 1.43) | 0.60(0.53, 0.65) |
| Central China | 1.28 | 0.65 | 66.26% | 33.74% | 2.15(1.93, 2.35) | 1.43(1.27, 1.56) | 0.73(0.65, 0.80) |
| Northeast China | 1.26 | 0.71 | 63.86% | 36.14% | 1.95(1.36, 2.37) | 1.25(0.83, 1.53) | 0.71(0.47, 0.87) |
| West China | 1.34 | 0.74 | 64.45% | 35.55% | 2.03(1.77, 2.20) | 1.31(1.14, 1.42) | 0.72(0.63, 0.78) |
| Multi-attribute groups |  |  |  |  |  |  |  |
| Lowest LE with unmet needs | 0.60 | 0.36 | 62.50% | 37.50% | 1.07(0.72, 1.28) | 0.67(0.45, 0.80) | 0.40(0.27, 0.48) |
| Highest LE with unmet needs | 2.00 | 1.12 | 64.18% | 35.82% | 3.40(2.99, 3.71) | 2.18(1.92, 2.38) | 1.22(1.07, 1.33) |

**Supplementary Table A4** Number of Missing Values in the Dataset

|  | Observations (N = 42480) | |
| --- | --- | --- |
|  | **Number of missing values** | **%** |
| States of care needs | 230 | 0.54 |
| Age | 0 | 0 |
| Gender | 0 | 0 |
| Marital status and living arrangements | 1128 | 2.66 |
| Rural-urban residents | 1280 | 3.00 |
| Education | 93 | 0.22 |
| Region | 0 | 0 |

**Supplementary Table A5** Comparing Estimated Total Life Expectancy for the Chinese Older Population

|  | Total Life Expectancy | |
| --- | --- | --- |
|  | **ven den Hout et al.’s (2019) approach** | **United Nations estimates** |
| Age |  |  |
| 60 | 21.4 | 21.3 |
| 65 | 17.4 | 17.3 |
| 70 | 13.8 | 13.7 |
| 75 | 10.6 | 10.4 |
| 80 | 7.9 | 7.6 |
| 85 | 5.8 | 5.4 |
| Male |  |  |
| 60 | 19.9 | 19.4 |
| 65 | 15.8 | 15.6 |
| 70 | 12.4 | 12.3 |
| 75 | 9.6 | 9.3 |
| 80 | 6.9 | 6.7 |
| 85 | 4.9 | 4.7 |
| Female |  |  |
| 60 | 23.2 | 23.2 |
| 65 | 19.0 | 19.0 |
| 70 | 15.1 | 15.0 |
| 75 | 11.7 | 11.5 |
| 80 | 8.8 | 8.3 |
| 85 | 6.4 | 5.9 |

**Supplementary Table A6** Life Expectancy (LE) with Unmet Care Needs among Chinese older people at the age of 60: Comparing Analyses of 50+ and 60+ CHARLS sample

|  |  | Overall | Men | Women |
| --- | --- | --- | --- | --- |
| LE with Unmet ADL Needs | Sample: People aged 50 and over | 0.70 | 0.48 | 0.96 |
|  | Sample: People aged 60 and over | 0.70 | 0.49 | 0.92 |
|  | **Difference** | 0.00 | -0.01 | 0.04 |
| LE with Unmet IADL Needs Only | Sample: People aged 50 and over | 1.35 | 0.99 | 1.75 |
|  | Sample: People aged 60 and over | 1.32 | 0.98 | 1.73 |
|  | **Difference** | 0.03 | 0.01 | 0.02 |
| LE with Unmet Needs | Sample: People aged 50 and over | 2.05 | 1.47 | 2.71 |
|  | Sample: People aged 60 and over | 2.02 | 1.47 | 2.65 |
|  | **Difference** | 0.03 | 0.00 | 0.06 |

**Supplementary Table A7** Logistic Regression Analysis of Association between Unmet Needs and Loss to Follow-up, CHARLS 2013-2020

| Independent variables | | Coefficient (standard error) |
| --- | --- | --- |
| Having unmet care needs | | 0.061(0.104) |
| IADL scores | | 0.010(0.016) |
| ADL scores | | 0.001(0.012) |
| Age groups (Ref: 60-69) | |  |
|  | 70-79 | 0.138^*^(0.063) |
|  | >=80 | 0.301^**^ (0.101) |
| Gender (Ref: Women) | | -0.006(0.058) |
| Education (Ref: Higher education) | | -0.465^***^(0.065) |
| Marital status & living arrangements (Ref: Married or cohabiting) | |  |
|  | Single & living with others | -0.098(0.113) |
|  | Single & living alone | -0.194^*^(0.094) |
| Rural-urban residents (Ref: Urban residents) | | -1.235^***^ (0.067) |
| Region (Ref: East China) | |  |
|  | Central China | -0.260^**^(0.075) |
|  | North China | 0.738^***^(0.100) |
|  | West China | 0.088(0.069) |

*Note:* Outcome variable: Loss to follow-up (coded as 1) vs survey participation (coded as 0); ^***^p<.001, ^**^p<.01, ^*^p<.05.

**Supplementary Table A8** Differences in Life Expectancy with Unmet Needs between Social Groups, People Aged 60 Years Old in China, Central Estimates and 95% Confidence Intervals

|  | **Differences** | **Lower limit** | **Upper limit** |
| --- | --- | --- | --- |
| **Women vs. Men** | | | |
| LE-Unmet IADL Needs Only | 0.751 | 0.545 | 0.958 |
| LE-Unmet ADL Needs | 0.435 | 0.328 | 0.542 |
| **Lower education vs. Higher education** | | | |
| LE-Unmet IADL Needs Only | 0.600 | 0.416 | 0.785 |
| LE-Unmet ADL Needs | 0.190 | 0.076 | 0.304 |
| **Single living with others vs. Married or cohabiting** | | | |
| LE-Unmet IADL Needs Only | 0.497 | 0.258 | 0.735 |
| LE-Unmet ADL Needs | 0.160 | 0.044 | 0.276 |
| **Single alone vs. Married or cohabiting** | | | |
| LE-Unmet IADL Needs Only | 0.494 | 0.230 | 0.758 |
| LE-Unmet ADL Needs | 0.334 | 0.179 | 0.488 |
| **Rural residents vs. Urban residents** | | | |
| LE-Unmet IADL Needs Only | 0.374 | 0.220 | 0.528 |
| LE-Unmet ADL Needs | 0.142 | 0.061 | 0.224 |
| **Central China vs East China** | | | |
| LE-Unmet IADL Needs Only | 0.115 | -0.090 | 0.321 |
| LE-Unmet ADL Needs | 0.056 | -0.044 | 0.155 |
| **Northeast China vs East China** | | | |
| LE-Unmet IADL Needs Only | -0.063 | -0.424 | 0.298 |
| LE-Unmet ADL Needs | 0.108 | -0.091 | 0.307 |
| **West China vs East China** |  |  |  |
| LE-Unmet IADL Needs Only | -0.002 | -0.201 | 0.197 |
| LE-Unmet ADL Needs | 0.124 | 0.023 | 0.224 |
| **Highest vs lowest LE with Unmet Needs** | | | |
| LE-Unmet IADL Needs Only | 1.515 | 1.222 | 1.808 |
| LE-Unmet ADL Needs | 0.818 | 0.649 | 0.986 |

*Note*: The difference (Group a vs Group b) is calculated as Group a minus Group b. Lowest LE with unmet needs: older people who were men, married or cohabiting, with higher levels of education, and living in urban areas; Highest LE with unmet needs: older people who were women, single, with lower levels of education, and living in rural areas.

**Supplementary Fig. A1** The Flow Chart of the CHARLS Samples (2013-2020) and Sample Selection Criteria

16,262

Continuing participants

18,612 Participants

42,480 Observations included in the analysis

Excluding observations with

·age below 60 years (n=38,170, 47.1%)

·missing age information (n=327, 0.4%)

80,977 Observations

in CHARLS 2013-2020

548

Died

1,802

Loss to follow-up

4,835

Refreshed samples

1,681

Refreshed samples

1,740

Refreshed samples

889

Died

620

Died

2,073

Loss to follow-up

1,541

Loss to follow-up

18,135

Continuing participants

17,655

Continuing participants

**Wave 2015**

**Wave 2018**

**Wave 2020**

**Wave 2013**

**Supplementary Fig. A2** The Measurement of Met and Unmet Care Need

Older people

aged 60+

Reported any care needs?

**Unmet care needs**

**Met care needs**

**No care needs**

Unmet IADL needs only

Received the corresponding help?

No

Yes

Received help with all needs

Didn’t receive help with at least one need

Unmet IADL & ADL needs

Which need?

IADL only

ADL only

IADL &ADL

Unmet ADL needs only

Unmet ADL needs

**Supplementary Fig. A3** Life Expectancy (LE) with Met and Unmet Care Needs According to the Number or Type of ADL and IADL Tasks

a) Life Expectancy with Met or Unmet Care Needs (years)

|  | LE-No Care Needs | LE-Met Care Needs | LE-Unmet Needs |
| --- | --- | --- | --- |
| Taking medication | 18.75 | 2.1 | 0.6 |
| Managing money | 18.2 | 2.5 | 0.74 |
| Shopping | 17.49 | 3.45 | 0.5 |
| Cooking | 17.19 | 3.46 | 0.8 |
| Doing housework | 16.7 | 3.34 | 1.39 |
| Using the toilet | 18.42 | 1.93 | 1.07 |
| Moving | 19.25 | 0.98 | 1.15 |
| Eating | 20.15 | 0.8 | 0.4 |
| Bathing | 18.12 | 2.39 | 0.89 |
| Dressing | 19.12 | 1.4 | 0.81 |
| At least Two ADL or IADL tasks | 14.94 | 6.03 | 0.4 |
| At least one ADL or IADL task | 14.98 | 4.42 | 2.02 |

b) Proportions of Life Expectancy with Met or Unmet Care Needs (%)

|  | LE-Met Care Needs/ LE-with Care Needs | LE-Unmet Needs/ LE-with Care Needs |
| --- | --- | --- |
| Taking medication | 77.7778% | 22.2222% |
| Managing money | 77.1605% | 22.8395% |
| Shopping | 87.3418% | 12.6582% |
| Cooking | 81.2207% | 18.7793% |
| Doing housework | 70.6131% | 29.3869% |
| Using the toilet | 64.3333% | 35.6667% |
| Moving | 46.0094% | 53.9906% |
| Eating | 66.6667% | 33.3333% |
| Bathing | 72.8659% | 27.1341% |
| Dressing | 63.3484% | 36.6516% |
| At least Two ADL or IADL tasks | 93.7792% | 6.22084% |
| At least one ADL or IADL task | 68.6335% | 31.3665% |
